# Structure function analysis of SH2D2A isoforms expressed in T cells reveals a crucial role for the proline rich region encoded by *SH2D2A *exon 7

**DOI:** 10.1186/1471-2172-7-15

**Published:** 2006-07-13

**Authors:** Stine Granum, Vibeke Sundvold-Gjerstad, Ke-Zheng Dai, Kristin Melkevik Kolltveit, Kjersti Hildebrand, Henrik S Huitfeldt, Tor Lea, Anne Spurkland

**Affiliations:** 1 Department of Anatomy, Institute of Basic Medical Sciences, Box 1105, Blindern, N-0317 Oslo, Norway; 2 Institute of Oral Biology, University of Oslo, N-0317 Oslo, Norway; 3 Institute of Pathology, Rikshospitalet University Hospital, N-0027, Norway; 4 Institute of Immunology, Rikshospitalet University Hospital, N-0027, Norway

## Abstract

**Background:**

The activation induced T cell specific adapter protein (TSAd), encoded by *SH2D2A*, interacts with and modulates Lck activity. Several transcript variants of TSAd mRNA exist, but their biological significance remains unknown. Here we examined expression of SH2D2A transcripts in activated CD4+ T cells and used the SH2D2A variants as tools to identify functionally important regions of TSAd.

**Results:**

TSAd was found to interact with Lck in human CD4+ T cells *ex vivo*. Three interaction modes of TSAd with Lck were identified. TSAd aa239–256 conferred binding to the Lck-SH3 domain, whereas one or more of the four tyrosines within aa239–334 encoded by SH2D2A exon 7 was found to confer interaction with the Lck-SH2-domain. Finally the TSAd-SH2 domain was found to interact with Lck. The SH2D2A exon 7 encoding TSAd aa 239–334 was found to harbour information essential not only for TSAd interaction with Lck, but also for TSAd modulation of Lck activity and translocation of TSAd to the nucleus. All five SH2D2A transcripts were found to be expressed in CD3 stimulated CD4+ T cells.

**Conclusion:**

These data show that TSAd and Lck may interact through several different domains and that Lck TSAd interaction occurs in CD4+ T cells *ex vivo*. Alternative splicing of exon 7 encoding aa239–334 results in loss of the majority of protein interaction motives of TSAd and yields truncated TSAd molecules with altered ability to modulate Lck activity. Whether TSAd is regulated through differential alternative splicing of the SH2D2A transcript remains to be determined.

## Background

When the human genome was sequenced, it was found to contain approximately 35 000 genes [1]. Considering the complex biological system encoded by these genes, this seems to be a small number. However, alternative transcripts that arise from different transcription start sites or alternative splicing, may yield a much larger array of functionally related proteins than suggested from the number of actual genes. Large scale genomic studies have revealed that 65% of all genes have alternative transcripts [2]. Elucidation of the role and regulation of transcript variants may shed light on how a rather modest number of genes can give rise to the complexity of human biology [2-4].

Related proteins encoded by the same gene through alternative transcription may have opposing functions in the cell. For instance, transcription factors may exist as isoforms lacking particular DNA- or protein interacting domains. When such isoforms are expressed, they may inhibit the function of the full length protein [5]. An *in silico *analysis of the mouse transcriptome revealed that alternative splicing preferentially adds or deletes domains in transcription factors that are important for DNA binding [6].

The function of a protein encoded by a particular gene may thus be regulated at the level of differential expression of various transcript variants dependent on the tissue or cell type, or the differentiation status and previous history of the cell [7]. One prominent example of this type of gene regulation in T cells, is the CD45 protein, a membrane receptor tyrosine phosphatase that exists as several isoforms generated by alternative splicing of exons [8]. The quality of the T cell response in naïve versus experienced T cells is determined in part by the composition of expressed CD45 isoforms on the cell surface [9-12].

Previously, we reported the cloning of the immunoregulatory gene *SH2D2A *that encodes the T cell specific adapter protein (TSAd) [13,14]. Adapter proteins lack catalytic activity, but may be crucial in regulation of cellular signalling by mediating protein-protein interactions through conserved protein binding domains [15]. The function of TSAd is as yet unclear, but the murine homologue Lad/RIBP has been identified as a binding partner for Lck, Itk/Rlk, MEKK2 and Grb2 [16-18] and human TSAd has been found to associate with Lck [19] as well as the molecular chaperone VCP [20] and vascular endothelial growth receptor 2 (VEGFR-2) [21]. Recently it has been shown that TSAd VEGFR-2 interaction promotes actin polymerization and migration [22]. TSAd may be involved in the regulation of membrane proximal T cell signalling by modulating the Lck activity [19,23,24], and it has been proposed that TSAd may be directly involved in regulation of gene transcription as a transcription adapter protein [25]. Mice lacking expression of TSAd are apparently normal [17], but as they grow older they spontaneously develop a systemic autoimmune disease [26].

Five naturally occurring transcript variants of human TSAd exist. The SH2D2A-1 (GeneBank: AJ000553) variant encodes full length TSAd [13], whereas the SH2D2A-2 (GenBank: AY763100) and -3 (GenBank:AY763098) variants encodes TSAd proteins shortened by 18 aa and 28 aa in the N-terminus, respectively. The SH2D2A-4 (GenBank:AY763099) variant encodes a 10 aa insertion in the SH2 domain of TSAd, whereas the SH2D2A-5 variant (GeneBank:AA063001) lacks the entire exon 7 [14], which encodes most of the proline rich region including the four C-terminal tyrosines (aa239–334) of TSAd. The SH2D2A-1-4 variants have been isolated from T cells, whereas the SH2D2A-5 variant was first identified in a pineal gland cDNA library [14].

In this study, we analysed TSAd molecules encoded by the SH2D2A transcript variants to identify potentially important functional domains. We identified TSAd aa239–334 encoded by exon 7, to be crucial for tyrosine phosphorylation of TSAd by Lck, for interaction with the Lck-SH2 and Lck-SH3 domain, and for modulation of Lck activity. In addition, TSAd aa239–334 controls TSAd translocation to the nucleus. In conclusion, this study demonstrates that exon 7 encodes structures of importance for TSAd function, and that truncated TSAd is co-expressed with the full length TSAd in CD4+ T cells. Whether alternative transcripts of TSAd contribute to regulation of TSAd function, by being differentially regulated under given circumstances or in given tissues remains to be elucidated.

## Results

### Expression of TSAd variants in primary CD4+ T cells

**Figure 1:**
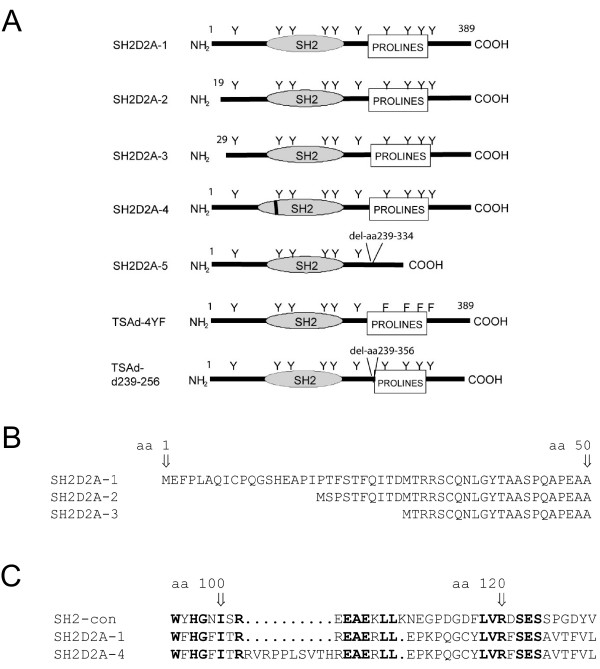
**An overview of the polypeptides encoded by SH2D2A transcript variants**. A. *Schematic presentation of putative protein-interacting domains in the SH2D2A isoforms and mutants described in this study: *The SH2D2A-1 encodes the full length TSAd, whereas the SH2D2A-2 and -3 variants represent different N-terminal sequences of TSAd. The SH2D2A-4 variant has a 10 aa insertion in the SH2-domain, whereas the fifth variant, SH2D2A-5, lacks aa239–334, containing the proline rich region including the four C-terminal tyrosines (Y = tyrosine). TSAd 4YF has the four C-terminal tyrosines exchanged with phenylalanine (F = phenylalanine). TSAd d239–256 has a deletion of amino acids 239–256 containing the motif PSQLLRPKPPIPAKPQLP. B. *Comparison of the N-terminal amino acid sequences of the SH2D2A-1, -2 and -3 variants: *The amino acid position is numbered according to full length TSAd (SH2D2A-1). The SH2D2A-2 and -3 variants differ from the SH2D2A-1 in the N-terminal 1–21 aa and 1–28 aa, respectively. C. *Comparison of the N-terminus SH2 domain aa sequences of the SH2D2A-1 and -4 variants: *The conserved arginine (R) at position 120 is marked. The SH2D2A-4 has an additional 10 aa (ins aa103–112) in the TSAd-SH2 domain. A SH2 consensus (con) sequence obtained from a Blast search, [42] is included for comparison. Conserved residues are marked in bold.

**Figure 2:**
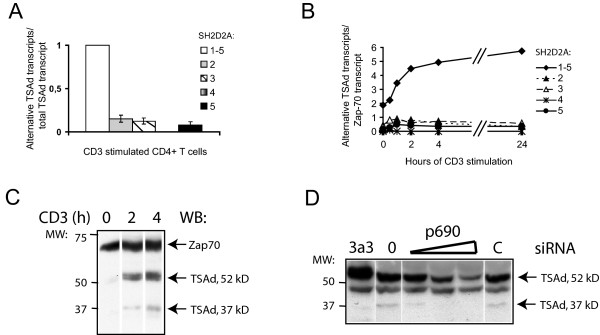
**Expression of SH2D2A variants in primary CD4+ T cells**. A. *Expression of SH2D2A transcript variants in anti-CD3 stimulated CD4+ T cells: *mRNA expression levels of SH2D2A variants relative to Zap-70 were assessed by RT-PCR in CD4+ T cells stimulated with CD3 ligation for 2 hours from four healthy blood donors. Results shown are median values ± SD of the estimated level of alternative SH2D2A transcripts relative to the total amount of SH2D2A transcripts. The SH2D2A-4 variant was not always observed. The median value of the SH2D2A-4 is 0,03% ± 0,2%. B: *The relationship between the different SH2D2A transcripts does not vary significantly throughout anti-CD3 stimulation of CD4+ T cells*: Primary CD4+ T cells were stimulated with anti-CD3 for 24 hours, and total amount of SH2D2A transcript (1–5) and mRNA levels of SH2D2A transcript variants 2, 3, 4 or 5 were assessed at different time points. After the initial two hours of anti-CD3 stimulation, the relationship between the five SH2D2A transcripts remained constant throughout the stimulation period. Results shown are median values of TSAd transcripts relative to Zap-70 transcripts. C: *TSAd of 37 kDa is expressed in CD3 stimulated primary CD4+ T cells*. Primary CD4+ T cells were stimulated with anti-CD3 for 2 and 4 hours and total sonicated cell lysates were separated by SDS-PAGE and immunoblotted with TSAd Abs and Zap-70 mAbs as a loading control. TSAd Abs detects two CD3 induced bands of 52 kDa and 37 kDa respectively. D. *Expression of TSAd of 52 and 37 kDa is repressed by siRNA treatment of CD4+ T cells: *Primary CD4+ T cells were transfected with control medium (0) or increasing concentration of TSAd siRNA (0,05, 0,5 or 5 μM p690) or with 5 μM control siRNA (C = TSAd p369) using Amaxa electroporator and stimulated with anti-CD3 beads for 24 hours. Total sonicated cell lysates were separated by SDS-PAGE and immunoblotted with TSAd Abs and Zap-70 mAbs as a loading control (not shown). Lysates of Jurkat TAg cells stably transfected with HA-tagged TSAd cDNA (3a3) was included as a blotting control. TSAd Abs detects two bands of 52 kDa and 37 kDa that can be inhibited by siRNA, and one non-specific band of 45 kDa, which is not affected by siRNA treatment.

Five transcript variants of TSAd that encode TSAd polypeptides with variable lengths have been reported (figure 1A–C). We previously observed that TSAd mRNAs SH2D2A-1-4 were expressed in PHA stimulated peripheral blood mononuclear cells [14]. In this study, we used reverse transcriptase quantitative PCR assay (RT-PCR) to assess the relative amounts of TSAd transcript variants expressed in primary CD4+ T cells (figure 2A). The SH2D2A-2 and -3 variants constituted 10–15% each, whereas the SH2D2A-5 variant which was originally cloned from a pineal gland library, constituted around 5–10% of the total amount of TSAd-transcripts in anti-CD3 stimulated CD4+ T cells from healthy blood donors. The SH2D2A-4 variant, although present, often fell below the detection limit of the assay. It is possible that the function of TSAd may be regulated by changing the relative amount of the different TSAd transcript variants. We therefore analysed the expression of TSAd transcript variants at different time points after anti-CD3 stimulation of CD4+ T cells. As seen from figure 2B, the relative distribution of the TSAd transcript variants was relatively constant during the observation period. Immunoblot of primary CD4+ T cells stimulated through ligation of CD3ε, revealed induction of a strong band of 52 kDa and a weaker band of 37 kDa reactive with TSAd antibody after 2 and 4 hours of stimulation. The 37 kDa band is induced in parallel with the 52 kDa TSAd reactive band (figure 2C) and is inhibited similar to TSAd in CD4+ T cells transfected with siRNA prior to CD3 stimulation (figure 2D). Taken together, these results suggest that different isoforms of TSAd are expressed in CD4+ T cells, and that TSAd in CD3 stimulated CD4+ T cells is not controlled through the differential expression of TSAd transcript variants.

### The TSAd N-terminus modifies phosphorylation of TSAd in Jurkat T cells

**Figure 3:**
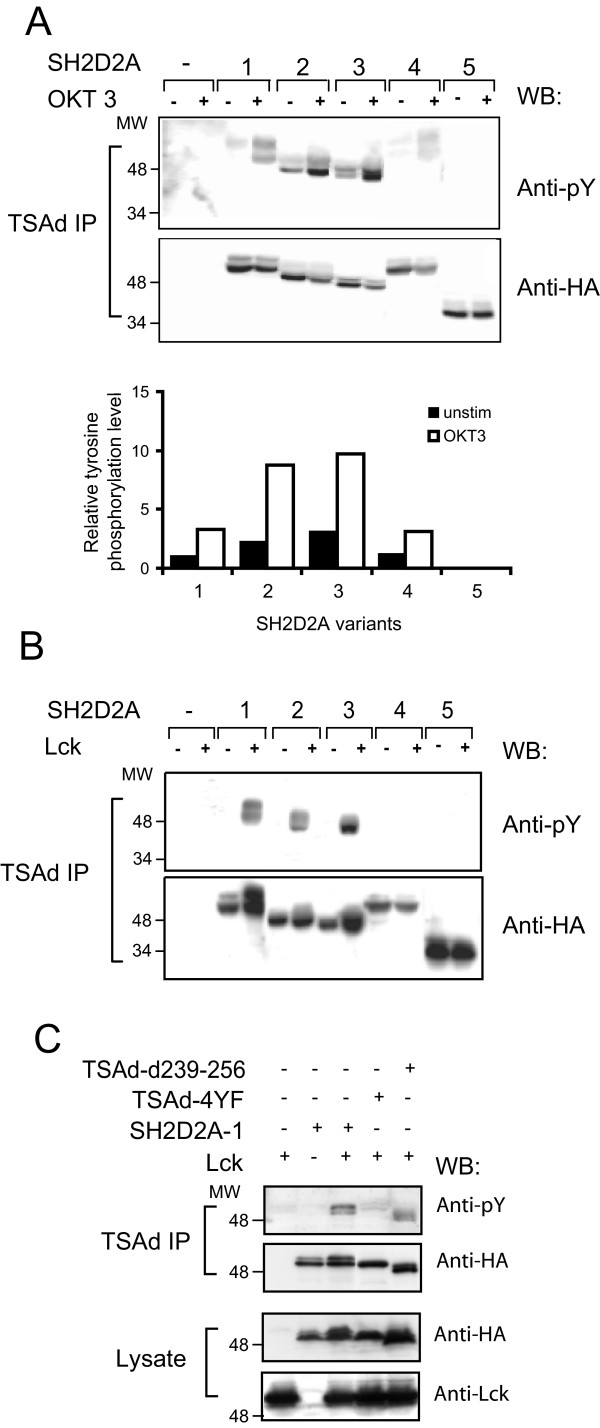
**Differential Lck phosphorylation of variant TSAd molecules**. A. T*yrosine phosphorylation of TSAd variants in Jurkat T cells: *Jurkat T cells transiently transfected with pEF-HA or pEF-HA-SH2D2A-1-5 cDNAs, were stimulated (+) with anti-CD3 (OKT3) mAbs for 2.5 min or left unstimulated (-), lysed and subjected to immunoprecipitation with anti-TSAd Abs (TSAd IP). The precipitates were separated by SDS-PAGE and immunoblotted with anti-phoshotyrosine (pY) mAbs (upper panel) and anti-HA mAbs (lower panel). Relative level of tyrosine phosphorylation of TSAd variants is shown in the chart, and the relationships between the pY signal versus the HA signal of bands is normalised to that observed for the full length TSAd IP (SH2D2A-1) expressed in resting Jurkat T cells. B. *Lck phosphorylates the SH2D2A-1, -2 and -3 variants of TSAd in 293T cells: *293T cells were transiently transfected with pEF-HA or pEF-HA-SH2D2A-1-5 cDNAs alone (-) or together (+) with pEF-Lck. The cells were lysed and treated as in A. C. *Tyrosine phosphorylation of TSAd mutated for the four C-terminal tyrosines is attenuated when co-expressed with Lck in 293T cells*. 293T cells were transiently transfected with pEF-HA, the pEF-HA-TSAd-4YF or pEF-HA-TSAd-d239–256 cDNAs together with pEF-Lck. The cells were lysed and treated as in A.

We then tested whether TSAd variants encoded by the *SH2D2A *gene displayed distinct functional properties. TSAd is phosphorylated upon tyrosines after CD3 stimulation [23] and Lck has been identified as a kinase that is able to interact with and phosphorylate TSAd [16,19]. We therefore assessed tyrosine phosphorylation of HA-tagged TSAd variants transiently expressed in Jurkat T cells before and after anti-CD3 stimulation. In comparison with full length TSAd (SH2D2A-1), TSAd molecules with shortened N-terminus (encoded by the SH2D2A-2 or 3 variants), displayed 2–3 fold higher levels of phosphorylation both in resting and CD3 stimulated Jurkat T cells (figure 3A). Both variants were tyrosine phosphorylated when co-expressed with Lck in 293T cells (figure 3B). These results suggest that the N-terminal 18 amino acids of TSAd harbours information that either modify TSAd interaction with Lck or other kinases within Jurkat T cells, or that the N-terminus contains some localisation signal, that when absent makes TSAd more accessible for particular tyrosine kinase activity within the Jurkat T cell.

### TSAd with aberrant SH2 domain is not phosphorylated by Lck

Several lines of evidence indicate that TSAd in addition to Lck may be tyrosine phosphorylated by other kinases, including Src [27], VEGFR-2 [22], PDGFR [28] and Zap-70 [19]. TSAd with an aberrant SH2 domain (encoded by SH2D2A-4) displayed a similar phosphorylation level as full length TSAd (i.e. the SH2D2A-1) when expressed in Jurkat T cells (figure 3A). We previously showed that truncated TSAd lacking the N-terminal aa 1–206 including TSAd SH2 domain, is phosphorylated by Lck when co-expressed in 293T cells [19]. However, surprisingly as shown in figure 3B, when expressed in 293T cells, Lck did not phosphorylate the SH2D2A-4 variant. This result is consistent with similar results obtained with TSAd mutated for the conserved arginine in position 120 in the SH2 domain (data not shown). Taken together, these results indicate that although the C-terminus of TSAd is sufficient for phosphorylation of TSAd by Lck, in non-T-cells there is a requirement for an intact SH2 domain for Lck mediated tyrosine phosphorylation of the full length TSAd.

### TSAd aa239–334 controls tyrosine phosphorylation of TSAd

TSAd lacking aa239–334 (SH2D2A-5) was not tyrosine phosphorylated in Jurkat T cells, nor in 293T cells when co-expressed with Lck (figure 3A and 3B). This indicates that aa239–334 harbors one or more tyrosine phosphorylation sites, or motifs crucial for interaction of TSAd with Lck or other tyrosine kinases. Indeed a Scansite search [29] identified several potential Lck-SH2 and Lck-SH3 interaction motifs within the TSAd aa239–334 [19]. To specifically test whether one or more of the four tyrosines in the aa239–334 sequence could be phosphorylated by Lck, we thus generated a TSAd mutant with all four tyrosines mutated to phenylalanine (TSAd-4YF). We also generated a TSAd mutant where a putative Lck-SH3 interaction site at aa239–256 was deleted (TSAd-d239–256). When transiently co-expressed with Lck in 293T cells TSAd-d239–256 was still phosphorylated on tyrosine, whereas TSAd-4YF was only very weakly phosphorylated (figure 3C).

### TSAd aa239–334 and the TSAd-SH2 domain mediate interaction with Lck

**Figure 4:**
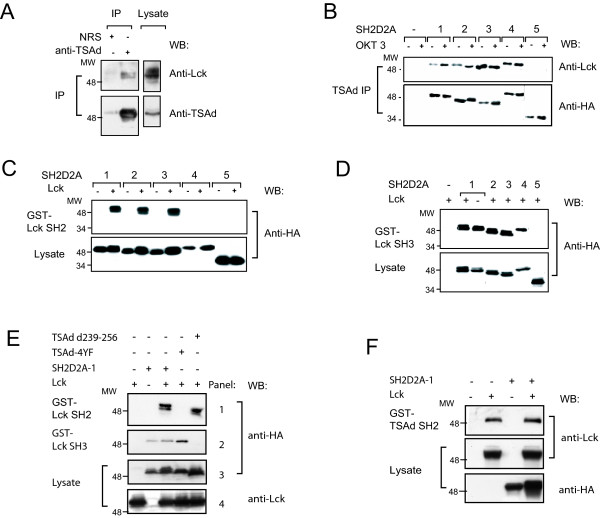
**SH2D2A exon 7 encodes ligands for Lck-SH2 and SH3 domains**. A. *TSAd interacts with Lck: *Primary CD4+ T cells were stimulated with anti-CD3/CD28 beads for one day. TSAd was precipitated with anti-TSAd Abs or irrelevant serum (NRS) and protein A/G sepharose beads from precleared lysates. Precipitates were separated by SDS-PAGE and immunoblotted with anti-Lck and anti-TSAd Abs as indicated. B. *TSAd variants interact with Lck in Jurkat T cells: *Jurkat T cells were transiently transfected with pEF-HA or one of the pEF-HA-SH2D2A-1-5cDNAs. Immunoprecipitation was performed using anti-TSAd Abs and protein G magnetic beads. Precipitates were separated by SDS-PAGE and immunoblotted with anti-Lck and anti-HA as indicated. C. *The SH2 domain and aa239–334 of TSAd is important for interaction with the Lck SH2 domain: *293T cells were transiently transfected with one of the pEF-HA-SH2D2A-1-5 cDNAs alone (-) or together (+) with pEF-Lck. Cell lysates were subjected to pull down experiment with GST-Lck-SH2 Sepharose beads. Precipitates were immunoblotted with anti-HA mAbs (upper panel). An anti-HA immunoblot of precleared lysates before GST-Lck-SH2 pull down is included to verify expression of the different HA-tagged TSAd variants (lower panel). D. *Aa239–334 contains ligands for the Lck-SH3 domain: *293T cells were transiently transfected with pEF-HA or one of the pEF-HA-SH2D2A-1-5 cDNAs together (+) with pEF-Lck. Only the pEF-HA-SH2D2A-1 cDNA were also co-transfected with pEF-HA (-). Cell lysates were subjected to pull down experiment with GST-Lck-SH3 Sepharose beads, and precipitates (upper panel) and precleared lysates (lower panel) were immunoblotted as in C. E. *Identification of TSAd structures interacting with Lck domains*: 293T cells were transiently transfected with pEF-HA, the pEF-HA-TSAd-4YF or pEF-HA-TSAd-d239–256 cDNAs together with pEF-Lck. Cell lysates were subjected to pull down experiment with GST-Lck-SH2 or GST-Lck-SH3 Sepharose beads. GST-Lck-SH2 (panel 1) and GST-Lck-SH3 (panel 2) precipitates were immunoblotted with anti-HA mAbs, and the precleared lysates were immunoblotted with anti-HA mAbs (panel 3) or anti-Lck mAbs (panel 4). F. *The SH2 domain of TSAd precipitates Lck in 293T cells: *293T cells were transiently transfected with pEF-HA, pEF-Lck, pEF-HA-SH2D2A-1 or pEF-Lck and pEF-HA-SH2D2A-1 cDNAs together. Precleared cell lysates were subjected to pull down experiment with GST-TSAd-SH2 Sepharose beads. Precipitates were immunoblotted with anti-Lck mAbs (upper panel). An anti-Lck immunoblot of precleared lysates before GST-TSAd-SH2 pull down is included to verify expression of Lck (lower panel).

We previously demonstrated that TSAd interacts with Lck in Jurkat T cells stably expressing HA-tagged TSAd [19]. Here we demonstrate that endogenous TSAd interacts with Lck in CD3/CD28 stimulated human peripheral blood CD4+ T cells (Figure 4A). In Jurkat transient transfectants, Lck co-precipitates with TSAd variants 1–4 (figure 4B). Upon longer exposure, a weak Lck band can also be seen in immunoprecipitates of TSAd lacking aa239–334 (not shown). In pull down experiments, Lck-SH2 domain binds to full length TSAd and TSAd with truncated N-terminus (SH2D2A-2 and -3) but only when co-expressed with Lck (figure 4C). Furthermore, the Lck-SH3 domain could precipitate all TSAd variants except for TSAd lacking aa239–334 (SH2D2A-5) (figure 4D). Similar pull down experiments using the TSAd-4YF and TSAd d239–256 mutants showed that Lck-SH3 domain interaction is dependent on the aa239–256, whereas the Lck-SH2 domain interaction is dependent on one or more of the four tyrosines within aa239–334 (figure 4E). Finally, and consistent with the weak co-immunoprecipitation of Lck seen with TSAd aa239–334, the TSAd-SH2 domain interacted with Lck in pull down experiments (figure 4F). Taken together, these data are in accordance with our previous observation that interaction of TSAd with the Lck SH2 and the Lck SH3 domains requires the C-terminus of TSAd (aa 189–aa 389) [19] and strongly suggests that the aa239–334 is of major importance for TSAd interaction with Lck *in vivo*. Furthermore, phosphorylation of TSAd by Lck is dependent on the presence of one or more of the four C-terminal tyrosines whereas the Lck-SH3 interaction site is dispensable.

### Tyrosines in TSAd aa239–334 confer the modulatory effect of TSAd on Lck activity

**Figure 5:**
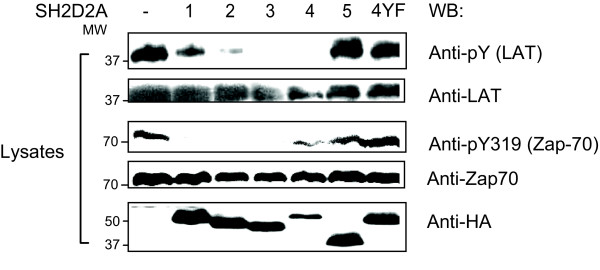
**Tyrosines within aa239–334 determines the inhibitory effect of TSAd on Lck activity**. The phosphorylation level of LAT and Y319 of Zap-70 in Jurkat T cells expressing the SH2D2A-5 variant and TSAd-4YF is normal upon TCR stimulation: Jurkat T cells were transiently transfected with either pEF-HA or one of the pEF-HA-SH2D2A-1-5 or pEF-HA-TSAd-4YF cDNAs. The cells were stimulated (+) with anti-CD3 (OKT3) mAbs for 2.5 min and lysed. The lysates were immunoblotted with Abs as indicated.

We have previously reported that TSAd inhibits early T cell signalling by inhibiting Lck activity [19,23]. Lck is the first kinase to become activated after triggering of the TCR. Phosphorylation of the ITAMs of CD3ζ recruits Zap-70 and Zap-70 Y319 becomes phosphorylated by Lck or Fyn [30,31]. Zap-70 thus activated subsequently phosphorylates the transmembrane adapter LAT [32]. Phosphorylation of Zap-70 Y319 and LAT in resting and stimulated T cells can thus be viewed as indirect measures of Lck activity within the cell. Immunoblots of cell lysates from CD3ε stimulated Jurkat cells transiently transfected with SH2D2A variants 1–4 showed decreased phosphorylation of Zap-70 Y319 and LAT, whereas in cells expressing the SH2D2A-5 variant or TSAd-4YF no change in Y319 or LAT phosphorylation was observed (figure 5). Taken together, these data show that the tyrosines in TSAd aa239–334 is essential not only for TSAd phosphorylation by and interaction with Lck, but also the modulating effect of TSAd on Lck activity.

### Nuclear localisation of TSAd depends on the TSAd-SH2 domain as well as aa239–334

**Figure 6:**
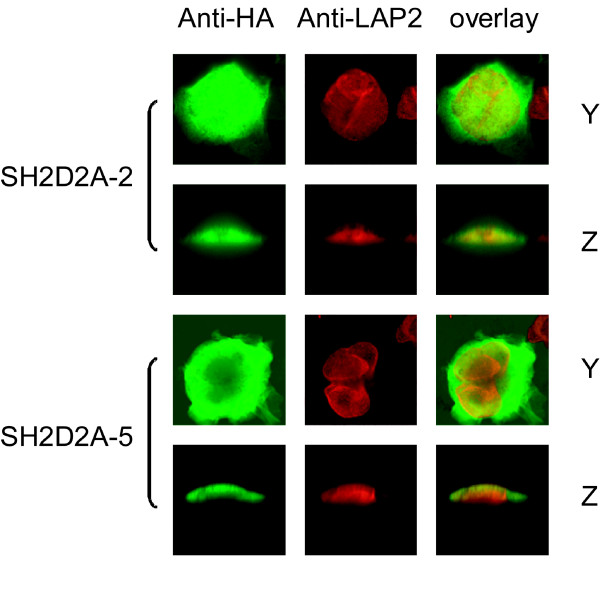
**Nuclear translocation of TSAd depends on aa239–334**. *The SH2D2A-5 variant is excluded from the cell nucleus in Jurkat TAg cells: *Confocal immunocytochemistry analysis of Jurkat TAg cells transiently transfected with pEF-HA-SH2D2A-2 and -5. The nuclear membrane protein LAP-2 was stained red (Cy™-3), whereas the HA-tagged recombinant proteins are stained green (Cy-2™). Cells were stimulated with anti-CD3 mAbs (OKT3) for 30 min. Horizontal sections are labelled Y, vertical sections are labelled Z (100× objective, 4× zoom).

The localisation of TSAd was initially found to be cytosolic [13], but was later also shown to be nuclear in activated T cells. TSAd was thus proposed to play a role in transcriptional regulation [25]. Here we used immunocytochemistry to assess the intracellular localisation of HA-tagged TSAd variants transiently expressed in Jurkat TAg cells. The nuclear membrane protein LAP-2 was used as a marker for the nuclear membrane. Similar to full length TSAd, N-terminally truncated TSAd variants were localised both in the cytoplasm and in the nucleus. In contrast, TSAd with disrupted SH2 domain or TSAd without aa239–334 were only found in the cytoplasm (figure 6 and data not shown). Thus nuclear localisation of TSAd depends not only on the TSAd-SH2 domain [25] but also on the TSAd C-terminal sequence aa239–334.

## Discussion

Alternative transcription may extend the repertoire of functionally different proteins expressed from one single gene. In this study, alternative transcript variants of SH2D2A were used as a tool to identify functionally relevant structures of TSAd. We found that TSAd variants displayed different functional features, indicating that differential expression of SH2D2A transcript variants may contribute to the regulation of TSAd function.

**Figure 7:**
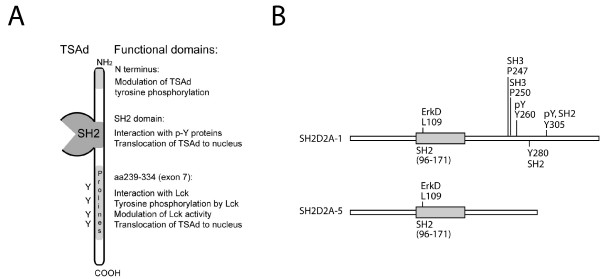
**Schematic presentation of TSAd functional domains and predicted sites**. A *Schematic presentation of functional regions of TSAd*. B *Predicted sites identified in high stringency Scansite search of SH2D2A-1 and SH2D2A-5 peptide sequences: *Core amino acids are indicated. pY: tyrosine kinase phosphorylation site. SH2 and SH3: SH2 and SH3 binding sites respectively. ErkD: binding site for ErkD domain.

Our findings are summarised in figure 7A. We revealed a prominent role for aa239–334 encoded by SH2D2A exon 7 in controlling TSAd interaction with Lck, modulation of proximal TCR signalling as well as translocation of TSAd to the nucleus, indicating that TSAd function may be regulated through alternative splicing of exon 7. In addition, the TSAd N-terminus and the SH2 domain affected TSAd phosphorylation by Lck.

In support of the notion that TSAd aa239–334 is important for TSAd function, a high stringency Scansite search [29] of possible protein interaction motifs in TSAd encoded by SH2D2A-1 revealed that several possible ligands for SH2 and SH3 domains, as well as tyrosine kinase substrates are encoded by exon 7 and are thus contained within aa239–334 (Figure 7B). A low stringency search also revealed one possible SH2 and several possible SH3 interaction sites encoded outside of exon 7 (not shown). However, a Scansite search is limited in that functionally relevant motifs may not appear in searches using the motif algorithms currently available. In particular, although our present studies indicate a role for the N-terminus in modulating TSAd tyrosine phosphorylation, no interaction motif is found by Scansite in the TSAd N-terminus. The motif database underlying Scansite searches are based on high affinity interactions as determined by selection of peptides from expression libraries. Recently, peptides representing all cytoplasmic phosphorylation sites of the four members of the EGFR receptor family were tested for binding to all SH2 domains in the genome using plasmon resonance [33]. In those instances where a consensus SH2 domain binding motif was available in Scansite, there were only partial overlap between the EGFR motives predicted from Scansite analysis and the EGFR motives found to bind to the recombinant SH2 domains [33]. It thus remains to be determined whether TSAd encoded by SH2D2A-5 is able to interact with other proteins in an SH2 domain independent manner.

Previous studies on TSAd and murine homologue LAD supports the notion that motives in the aa239–334 are functionally important. We previously reported that Lck activity is modulated by the C-terminus of TSAd [19], and Choi et al reported that the isolated LAD-SH2 domain is unable to modulate Lck activity [16]. Recently Marti et al reported that both the three C-terminus LAD tyrosines and a predicted Lck-SH3 interaction site in the C-terminal of LAD influenced LAD modulation of Lck activity [24]. Moreover, Grb2 interacts with LAD Y292 (i.e. TSAd Y280), and Y292 and Y302 (i.e. TSAd Y290 and Y305) mediates PDGF induced AP-1 transcriptional activity [28]. Thus, taken together these previous studies as well as the present study strongly point to a role for TSAd aa239–aa334 in control of various functions of TSAd.

Differential regulation of the expression of functionally distinct alternate transcripts from a single gene may contribute to the regulation of the gene's function. We found that the alternative TSAd transcripts may constitute up to 15–30% of the total amount of SH2D2A mRNA in the CD4+ T cell. At the protein level, we showed that in CD4+ T cells, although the full length TSAd 52 kDa band dominate, also TSAd of 37 kDa lacking the aa239–334 is expressed. However, we did not reliably observe protein bands representing N-terminally truncated TSAd variants. When expressed in Jurkat T cells or 293T cells, each of the SH2D2A cDNAs produced TSAd molecules of expected lengths (figure 3A and 3B and 4B–D), indicating that all TSAd variant mRNAs encoded by the plasmid constructs were also translated. When co-transfecting Jurkat T cells with SH2D2A-1 cDNA together with 10% of the amount of SH2D2A-3 cDNA, we were not able to detect the SH2D2A-3 protein band on Western blots, although the SH2D2A-3 cDNA when transfected alone did reveal a distinct protein band of expected length (data not shown). This indicates that the N-terminally truncated TSAd molecules fall below the detection limit of global TSAd Western blot analysis. In order to directly assess the expression of variant TSAd molecules antibodies specifically recognizing one but not any of the other TSAd molecules must be generated. However, the sequence differences between the various TSAd molecules are minimal, and the predicted antigenicity as assessed by the program "antigenic" of the EMBOSS suite [34,35] of the N-terminal TSAd amino acids, as well as the exon 6–8 boundary is not favourable. At present it is therefore not possible to determine to what extent these transcript variants are also represented at the protein level.

We were not able to demonstrate differential regulation of the different alternate transcripts of the *SH2D2A *gene in anti-CD3 stimulated CD4+ T cells. However, we have only examined expression of TSAd transcripts in one cell type under one stimulatory regimen. Physiological T cell activation also requires the presence of co-stimulatory signals delivered through CD28-B7 interactions. In addition, TSAd mRNA expression may be induced in CD4+ T cells via cAMP dependent signals [36]. Since the initial cloning and characterisation of TSAd [13,17], TSAd has also been found to be expressed in endothelial [21,22] and epithelial cells [28]. It is possible that the pattern of SH2D2A transcripts differ in these cells.

The importance of the SH2 domain for nuclear translocation of TSAd was previously pointed out by Marti et al [25]. Here we extend these data by showing that also the aa239–334 of TSAd is necessary for translocation of TSAd to the nucleus. TSAd does not contain a nuclear localisation signal, but are associated with the molecular chaperone Valocin-containing protein/p97 (VCP), and this interaction is thought to be necessary for translocation of TSAd to the nucleus [20]. How TSAd interacts physically with VCP is not yet known. The isolated TSAd-SH2 domain has been shown to be translocated to the nucleus [25]. It is however conceivable that the intact TSAd molecule may have different requirements for interaction with VCP and translocation to the nucleus, as indicated by our observation that truncated TSAd with intact SH2 domain fails to be translocated to the nucleus.

The 10 aa insertion in the SH2 domain of the TSAd protein encoded by the SH2D2A-4 cDNA probably result in a misfolded SH2 domain. This variant behaved like TSAd harbouring *in vitro *generated point mutation of the conserved arginine in the SH2 domain (R120K) ([19,25] and data not shown) supporting this notion. The SH2D2A-4 transcript was not consistently observed in quantitative PCR analysis of anti-CD3 stimulated CD4+ T cells, and it is thus probably expressed at very low levels in T cells. Taken together, this indicates that the SH2D2A-4 transcript has no functional relevance in the cell and probably represents transcriptional noise.

## Conclusion

We have demonstrated functional differences between TSAd molecules encoded by alternative transcript variants of the *SH2D2A *gene, and these studies have pointed out a crucial role for aa239–334 encoded by exon 7 in mediating TSAd function (figure 7). Further studies are needed to assess to what extent differential regulation of the alternate SH2D2A transcripts may contribute to the regulation of TSAd function in T cells and other cell types.

## Methods

### Plasmids

The TSAd variants SH2D2A-1-4 were isolated from a cDNA library from activated CD8+ T cells [13], and cloned into the EcoRI site of the mammalian expression vector pEF/HA. The SH2D2A-5 variant was derived from an EST clone (IMAGE clone id: 382223, GeneBank:AA063001). Since this clone contains a partial cDNA, the cDNA was fused using mega PCR [37] with the lacking 5' end of the SH2D2A-1 variant. cDNA encoding the Lck-SH3 domain as well as the TSAd-SH2 domain were subcloned into the pGEX-6P-1 expression vector (Amersham Biosciences, Uppsala, Sweden). The pEF-Lck and pGEX-3T-Lck SH2 constructs were generous gifts from Dr. Tomas Mustelin.

### Antibodies

The following monoclonal antibodies (mAbs) were used: anti-Zap-70 (Transduction Laboratories, Lexington, KY), anti-HA (Babco, Richmond, CA), anti-human CD3ε (OKT3, American Type Culture collection, Manassas, VA), anti-phosphotyrosine (clone 4G10, Upstate Biotechnology, Lake Placid, NY), anti-Lck (clone IF6, a generous gift from Joseph B. Bolen) and anti-LAP 2 (clone 27, Transduction Laboratories). The following polyclonal antibodies were used: anti-HA, anti-LAT and anti pY319 Zap-70 (Santa Cruz Biotechnology, Santa Cruz, CA), and anti-TSAd antibodies raised against synthetic peptides of TSAd [13,19]. Secondary antibodies were horseradish peroxidase-conjugated goat anti-mouse IgG, goat anti-rabbit IgG, donkey anti-rabbit IgG with fluorescence Cy™-2 and donkey anti-mouse IgG with fluorescence Cy™-3 (Jackson ImmunoResearch Laboratories, West Grove, PA).

### Cell cultures and transfections

Primary resting CD4+ T cells were positively selected from peripheral blood samples from healthy blood donors as described [38] using anti-CD4 coated Dynabeads (Dynal, Oslo, Norway). Jurkat TAg cells [39], 293T cells and Jurkat E6.1 cells (American Type Culture Collection) were cultured in RPMI 1640, 5–10% foetal calf serum (FCS) supplemented with 1 mM sodium pyruvate, 1 mM non-essential amino acids (all from GIBCO BRL^®^, Life Technologies™, The Netherlands) and antibiotics. Transfections of 2 × 10^7 ^Jurkat T cells in RPMI 1640 with 5% FCS with 5–30 μg plasmid DNA were performed using a BTX electroporator (Genetronix, San Diego, CA) at 200 V, 70 ms. Transfectants were cultured for 16–24 hours. 293T cells were transfected by Lipofectin (Invitrogen) in Optimem 1 medium (GIBCO BRL^®^) as described by the manufacturer.

### SiRNA-mediated attenuation of TSAd expression

SiRNA against the human TSAd sequence p690 5'-GGA CCG AAG AAU CAA ACU U tt-3', antisense 5'-AAG UUU GAU UCU UCG GUC C tc-3' (Eurogentec S.A.), was also previously described [22]. SiRNA directed towards human TSAd sense p369 5'-UGG UUC CAU GGC UUC AUC A tt-3' and antisense 5'-UGA UGA AGC CAU GGA ACC A tt-3' (Eurogentec S.A.), was used as a negative control since it was found unable to knock-down TSAd expression (data not shown). Transfection of 5 × 10^6 ^CD4+ T cells with 0.05–5 μM SiRNA were performed using the Amaxa electroporator [40] and Human T cell Nucleofector™ Kit (VPA-1002) following the description by the manufacturer.

### Cell stimulation, lysis, immunoprecipitation and Western blot

Primary CD4+ T cells were stimulated using CD3 Dynabeads (Dynal) or CD3/CD28 beads (Dynal) when indicated. For immunoprecipitation (IP) experiments, Jurkat T cells (1 × 10^7 ^cells/IP) were washed in RPMI 1640, resuspended and stimulated with 5 μg/ml anti-CD3 (OKT3) mAbs pr 5 × 10^7 ^cells for 2.5 min. Cells were lysed by adding an equal volume of 2 × lysis buffer (1 × lysisbuffer: 1% Nonidet P40 (Calbiochem-Novabiochem Corporation, La Jolla, CA) or 1% Igepal (Sigma-Aldrich, St. Louis, MO), 50 mM n-octyl-β-d-glucoside (Sigma-Aldrich), 20 mM Tris (pH 7.5), 100 mM NaCl, 50 mM NaF, 1 mM Na_3_VO_4_, 10 μg/ml of the protease inhibitors; leupeptin, pepstatin A, chymostatin and antipain (all from Sigma Aldrich). The 293T cells, Jurkat T cells or primary CD3/CD28 stimulated CD4+ T cells were washed with PBS and lysed in 1 × lysis buffer. Lysates were precleared 2–3× for 45 minutes with protein A/G Sepharose™ (Amersham Pharmacia Biotech, Uppsala, Sweden) or protein G magnetic beads (Dynalbiotech, Oslo, Norway), incubated with the relevant antibodies for 45 minutes followed by protein A/G or protein G beads precipitation for 1 hour. Precipitates were washed 3× in 1 × lysis buffer. Primary CD3 stimulated CD4+ T cells were sonicated in 1 × lysis buffer. Immunoprecipitations or cell lysates were separated by 8–10% SDS-PAGE and blotted onto a polyvinylidine difluoride membrane (PVDF) (BioRad Laboratories, Hercules, CA). Blots were probed with the indicated antibodies in Tris buffered saline (TBS, pH 7.4) with 0,1% Tween (Sigma Chemical, St. Louis, MO) and 3% bovine serum albumin (BSA) (Biotest, Dreieich, Germany) or 3% skimmed milk (Sigma-Aldrich). Binding of antibodies to the target proteins were detected by secondary HRP-labelled antibodies and Super Signal^® ^West Pico Stable Peroxide Solution (Pierce, Rockford, Illinois), using either Kodak BioMax MR film (Kodak, Rochester, NY) or Kodak Image Station 2000R (Kodak). Signal intensity of blots was analysed using Kodak 1D image Analysis software.

### GST-pull down assays

The GST-fusion proteins were produced in BL21 Codon plus bacteria (Stratagene, La Jolla, CA), and purified on glutathione Sepharose beads (Amersham Biosciences). Cellular lysates prepared as described above were precleared 3× for 1 hour, each with a 1:1 mixture of GST-glutathion/4B Sepharose. Precleared lysates of 5–10 × 10^6 ^cells, were added to aliquots of Lck-SH2, Lck-SH3 or TSAd-SH2-GST-gluthatione Sepharose beads and rotated for 1 hour. Beads were washed 3× with 1 × lysis buffer, proteins were eluted in SDS-loading buffer and separated on a 8–10% SDS-PAGE gel.

### Quantitative RT-PCR of SH2D2A transcripts

Total RNA were extracted using Absolutely RNA kit (Stratagene, La Jolla, Ca, USA) according to the manufacturers instructions. cDNA was synthesised from total mRNA using random nonamers and MMLV reverse transcriptase as described by the manufacturer (Eurogentec, Seraing, Belgium).

Quantitative real-time PCR and subsequent data analysis were performed using the Mx4000 Quantitative PCR system (Stratagene) equipped with version 4.0 software. The custom made primers and probes specific for TSAd transcripts and Zap-70 are shown in table 1 were ordered from Eurogentec. Primers and probes for TSAd universal and Zap-70 were designed by Primer Express (PE Applied Biosystems, Foster City, CA) whereas the primers and probes for the different splice variants were designed by us (table 1). None of the amplicons exceeded 150 bp in length. All probes had a darquencher. For TSAd universal- and Zap-70 primer/probe set, the PCR reaction contained 900 nM of each primer, 300 nM of the probe and 1 × TaqMan^® ^Universal PCR Master Mix (Applied Biosystems). For the TSAd transcript specific primer/probe sets, each PCR reaction contained 600 nM of the primers and 200 nM of the probe as well as 1 × TaqMan^® ^Universal PCR Master Mix. DNA amplification was performed with the following thermal cycling profile: initial incubation at 50°C for 2 minutes, initial denaturation at 95°C for 2 minutes, 40 cycles of amplification (denaturation at 95°C for 10 seconds and annealing and elongation for 1 minute at 60 or 62°C). Fluorescence data were collected during the annealing stage of amplification.

**Table 1:** Primers and probes for quantitative PCR

Oligo	Sequence	Specificity	GeneBank:	Position
19-FW	5'-CAT AGT TCC TCT CTG AGA AAC	SH2D2A-3	AY763098	7–27
WT-42-FW	5'-TCA TGG AGT TCC CCC TGG C	SH2D2A-2	BI820427	19–38
WT-42-19-RV	5'-AGC TCC TGC GGG TCA TGT	SH2D2A	AJ000553	169–186
45-FW	5'-CTG CCT GGT TCC ATG GCT T	SH2D2A	AJ000553	365–383
45-RV	5'-TCC AGC AGC CTC TCT GCC	SH2D2A	AJ000553	398–415
Ex7-FW	5'-CCA GTA CAG CCC AAT CAT CAA	SH2D2A	AJ000553	728–748
Ex7-RV	5'-TTG CCC AAT CAC AGA GTT CTC A	SH2D2A	AJ000553	1115–1136
ZAP-70-FW	5'-ACA CCC TCA ACT CAG ATG GAT ACA	Zap 70	BC053878	1036–1060
ZAP-70-RV	5'-TCG GCC GCG GTT TGT	Zap 70	BC053878	1090–1105
Probe-45	5'-FAM-TTC GGC CCC CTC TCT CCG TCA CC-DQ	SH2D2A-4	AY763100	397–419
Probe-WT-19	5'-FAM-GTC ACG AAG CCC CCA TCC CA-DQ	SH2D2A-1,3–5	AJ000553	124–144
Probe-42	5'-FAM-AGA TAT GTC CCC AAG CAC CTT CCA GAT C-DQ	SH2D2A-2	BI820427	41–77
Probe-ex7	5'-FAM-AGA AGG AGA ATA CAG GTG GCT CCC AGC-DQ	SH2D2A-5	AA063001	482–509
Probe Zap-70	5'-HEX-CCC TGA GCC AGC ACG CAT AAC GT-DQ	Zap-70	BC053878	1062–1184

A standard curve in triplicate was constructed for each transcript variant of TSAd and Zap-70. Serial dilutions of plasmids containing an insert with either the relevant TSAd transcripts or Zap-70 were made containing 10000, 1000, 100 and 10 fg/μl of the plasmid. Quantity of TSAd was estimated relative to the quantity of Zap-70. The choice of Zap-70 as a housekeeping gene was based on our previous studies which have shown that the amount of Zap-70 is relatively stable during the various phases of T cell activation [23,41]. Experiments were performed in duplicate for each data point.

## Authors' contributions

SG carried out most of the molecular genetic studies, and drafted the manuscript. VSG carried out part of the molecular genetic studies. KZD carried out the immunocytochemistry and the fluorescent microscopy. HSH carried out confocal microscopy. KMK carried out the quantization of alternative transcripts. KH carried out analysis of TSAd-SH2-Lck interaction. TL participated in the design of the study. AS conceived of the study, carried out part of the cloning and sequencing of constructs and participated in its design and coordination. All authors read and approved the final manuscript.
